# Scrotal Botulinum Toxin Exposure and Male Fertility: Mechanistic Insights and Clinical Evidence

**DOI:** 10.7759/cureus.105904

**Published:** 2026-03-26

**Authors:** Amta Azeem, Aviral C Sharma, Jose M Garchitorena, Adam Boukind, Ibrahim H Omari, Noah Nigro

**Affiliations:** 1 Medicine, Ponce Health Sciences University, St. Louis, USA; 2 Medicine, Washington University School of Medicine, St. Louis, USA; 3 General Practice, Sheba Medical Center, Ramat Gan, ISR; 4 Department of Surgery, Division of Urology, University of Arizona, Tucson, USA

**Keywords:** botulinum (botox), cremaster muscle, dartos muscle, male fertility, onabotulinumtoxina, scrotal botulinum toxin type a, scrotal rejuvenation, scrotox, spermatogenesis, testicular thermoregulation

## Abstract

Introduction: Scrotal botulinum toxin type A (BoNT-A) injections (“scrotox”) are increasingly used to relax the dartos and/or cremaster muscles for cosmetic purposes and for functional indications such as chronic scrotal pain/orchialgia, exaggerated cremasteric reflex, and cremasteric synkinesia. Evidence remains limited and dispersed across animal toxicology studies, urologic case reports, and pain-management trials. We conducted a systematic review to evaluate whether botulinum toxin exposure involving the scrotum, dartos, cremaster, spermatic cord/peritesticular region, or predefined regulatory male fertility datasets is associated with changes in male reproductive outcomes.

Methods: We performed a systematic review in accordance with PRISMA 2020. PubMed/MEDLINE, Embase, and Scopus were searched from inception through January 5, 2026, with additional identification via reference screening and targeted retrieval of publicly available regulatory reproductive-toxicity documentation for onabotulinumtoxinA and prabotulinumtoxinA, the products for which relevant public regulatory datasets were available. Eligible studies included animal experiments and human clinical reports involving relevant anatomic exposure sites and reporting at least one fertility-relevant outcome (e.g., sperm parameters, spermatogenesis-related histology, reproductive hormones, or fertility indices). Given heterogeneity in exposure routes, dosing, and outcomes, meta-analysis was not planned; results were synthesized qualitatively. Risk of bias was assessed using SYRCLE (animal studies), Joanna Briggs Institute tools (case reports/series), and RoB 2.0 (randomized trials), and evidence levels were categorized using Oxford CEBM.

Results: The search identified 93 records; after deduplication, 75 underwent title/abstract screening, 15 were reviewed in full text, and 10 studies met inclusion criteria for qualitative synthesis. Evidence was heavily weighted toward animal data. Animal studies provided moderate-quality evidence suggesting dose- and site-dependent reproductive effects: repeated or higher-dose exposures delivered adjacent to the testes (particularly intracremasteric administration) were associated with impaired spermatogenesis and adverse testicular histopathology, whereas very low-dose systemic intramuscular exposure distant from the testes was reported to be neutral or potentially beneficial in an ageing model. Human evidence was uniformly low or very low quality for fertility inference because published case reports, case series, and clinical trials primarily evaluated pain relief, neuromuscular outcomes, or cosmetic satisfaction and did not prospectively measure semen parameters, endocrine function, thermoregulatory metrics, or fertility endpoints.

Conclusions: Current evidence is insufficient to determine the reproductive safety of scrotal or peritesticular botulinum toxin injections in humans. Preclinical data raise concern for potential spermatotoxicity with higher-dose and/or anatomically proximal exposure, while human studies remain indirect and lack fertility-specific monitoring. Standardized dosing and technique reporting, incorporation of semen and hormonal outcomes, direct thermophysiological assessment, and longitudinal follow-up are needed. Until such data exist, clinicians should counsel men desiring future fertility that mechanistic risk is plausible and definitive human fertility outcomes have not been established.

## Introduction and background

Scrotal botulinum toxin injections, informally referred to as “scrotox,” have gained increasing attention as a minimally invasive procedure intended to relax the dartos muscle and improve scrotal aesthetics and comfort [1]. Scrotox injections were initially described to manage chronic orchialgia and exaggerated cremasteric reflexes [2], but have become popular for cosmetic purposes such as smoothing scrotal wrinkles and producing a more relaxed scrotal appearance. Beyond cosmetic use, clinicians have become increasingly interested in the potential functional implications of this intervention, particularly its possible impact on testicular thermoregulation [1,3]. Because spermatogenesis is highly temperature-sensitive, any therapy that alters scrotal tone or position may theoretically influence male reproductive physiology [4]. This emerging intersection between aesthetic urology and male fertility has prompted clinicians and researchers to evaluate scrotal botulinum toxin injections through a broader reproductive-health lens [5].

Botulinum toxin type A (BoNT-A) acts by inhibiting acetylcholine release at neuromuscular junctions, producing temporary muscle relaxation lasting several months [5,6]. The dartos muscle, a thin layer of smooth muscle in the scrotal wall, contracts to reduce surface area and conserve heat, while the cremaster muscle elevates the testes in response to cold or stress [7]. When injected into the scrotal dartos muscle, BoNT-A can reduce superficial smooth-muscle contractility, potentially lowering scrotal tightness and altering the temperature environment surrounding the testes [4,5]. Previous research has demonstrated that even subtle scrotal temperature elevations can negatively affect sperm concentration, motility, and morphology [8,9]. Consequently, therapeutic interventions that modify scrotal musculature require careful evaluation to determine whether they might inadvertently influence fertility outcomes.

Despite growing clinical interest, evidence on the reproductive consequences of scrotal botulinum toxin injections remains limited and scattered across cosmetic literature, urologic case series, and animal studies. Several early investigations have suggested that BoNT-A may play a role in modulating testicular blood flow and local tissue dynamics, although findings have been inconsistent and often derived from small samples [10,11]. The lack of standardized dosing protocols, variable injection techniques, and short follow-up durations further complicates the interpretation of available data [11]. As a result, clinicians currently lack evidence-based guidance to counsel patients who seek cosmetic scrotox but also hope to preserve or optimize fertility [1].

Emerging reports suggest that men undergoing scrotox are often younger [1,12]. There are currently no fertility-specific guidelines for scrotal botulinum toxin injections. Accordingly, this systematic review aims to synthesize existing preclinical and clinical evidence to identify knowledge gaps, assess reported outcomes and safety considerations, and clarify future research priorities related to male fertility. As the use of this procedure expands and patient concerns regarding reproductive implications increase, such synthesis is essential to support evidence-informed clinical decision-making and guide future investigation in aesthetic and reproductive urology.

## Review

Methods

Review Design and Reporting

We conducted a systematic review to evaluate whether botulinum toxin exposure involving the scrotum, dartos, cremaster, spermatic cord/peritesticular region, or predefined regulatory male fertility datasets is associated with changes in male reproductive biology, including sperm parameters, spermatogenesis, testicular histopathology, reproductive hormones, and fertility indices. The review was reported in accordance with the Preferred Reporting Items for Systematic Reviews and Meta-Analyses (PRISMA) 2020 statement. Given substantial heterogeneity in exposure routes, doses, and outcome measures, quantitative meta-analysis was not planned; findings were synthesized narratively.

Data Sources and Search Methods

We searched PubMed/MEDLINE, Embase, and Scopus from database inception through January 5, 2026 (final search date). Additional records were identified by screening reference lists of included studies and relevant publications, as well as through targeted retrieval of publicly available regulatory reproductive-toxicity documentation for onabotulinumtoxinA (BOTOX®; U.S. Food and Drug Administration) and prabotulinumtoxinA (Nuceiva®; European Medicines Agency). The full, exact, and reproducible search strategies for all databases are provided in Appendix A.

Record Management and Deduplication

All database results were exported on January 5, 2026, in RIS format and imported into EndNote 20. Duplicates were removed using EndNote’s “Find Duplicates” function, matching on title, year, and author, followed by manual adjudication to resolve near-duplicates (e.g., punctuation differences or Epub-ahead-of-print versus print records). The deduplicated library was then exported to a screening spreadsheet for title and abstract screening.

Eligibility Criteria

Studies were considered eligible if they reported original data from animal experiments, randomized trials, observational studies, case series, or case reports involving human males or male mammalian models. Eligible exposures included direct administration of botulinum toxin to the scrotal wall or dartos, cremaster muscle, peritesticular region, or spermatic cord/perispermatic cord region (including cord blocks performed at or near the external inguinal ring). Studies reporting predefined regulatory reproductive-toxicity datasets from the FDA or EMA for onabotulinumtoxinA or prabotulinumtoxinA were also eligible. In addition, systemic or distant intramuscular botulinum toxin exposure was included only when male reproductive outcomes-such as sperm parameters or testicular histology-were measured as primary study endpoints. To be eligible, studies were required to report at least one fertility-relevant outcome, including sperm characteristics, spermatogenesis-related histology, reproductive hormone measures, or fertility indices, and to be published in English. We excluded reviews, commentaries, and technique-only articles without original outcome data; conference abstracts without full text; studies limited to penile-only or pelvic-floor-only injections without scrotal, cremasteric, or peritesticular exposure; and botulinum toxin studies unrelated to male reproductive outcomes that did not meet the predefined regulatory dataset criterion.

Study Selection

Two reviewers independently screened titles and abstracts, followed by full-text review, using the predefined eligibility criteria. Disagreements were resolved by consensus. The study selection process is summarized in a PRISMA flow diagram.

Data Extraction

For each included study, we extracted data on study design; setting; model or population characteristics (including species, strain, and age for animal studies and clinical indication for human studies); botulinum toxin formulation, serotype, and product where available; dose, dilution, frequency, and injection site; duration of follow-up; fertility-relevant outcomes (including sperm count, motility, morphology, and maturation indices; testicular histology or pathology; endocrine markers when reported; and fertility indices such as mating success, conception rates, or litter parameters); and reported adverse events.

Risk-of-Bias Assessment

Risk of bias was assessed using design-appropriate tools. Animal studies were evaluated using the SYRCLE Risk-of-Bias tool for animal intervention studies [13]. Case reports and case series were assessed using the Joanna Briggs Institute (JBI) critical appraisal checklists [14]. Randomized controlled trials were assessed using the Cochrane Risk of Bias 2.0 tool [15]. Levels of clinical evidence were categorized using the Oxford Centre for Evidence-Based Medicine (CEBM) framework [16].

Data Synthesis

Given heterogeneity in exposure routes, dosing strategies, and reported outcomes, results were synthesized qualitatively. Findings were organized by evidence domain: (1) animal toxicology and mechanistic studies; (2) human functional, pain-related, and cosmetic reports; and (3) regulatory reproductive-toxicity evidence. Results were interpreted in relation to botulinum toxin dose, anatomic proximity to the testes, and the directness of fertility-related endpoints.

Results

Study Selection and Quality of Evidence

**Figure 1:**
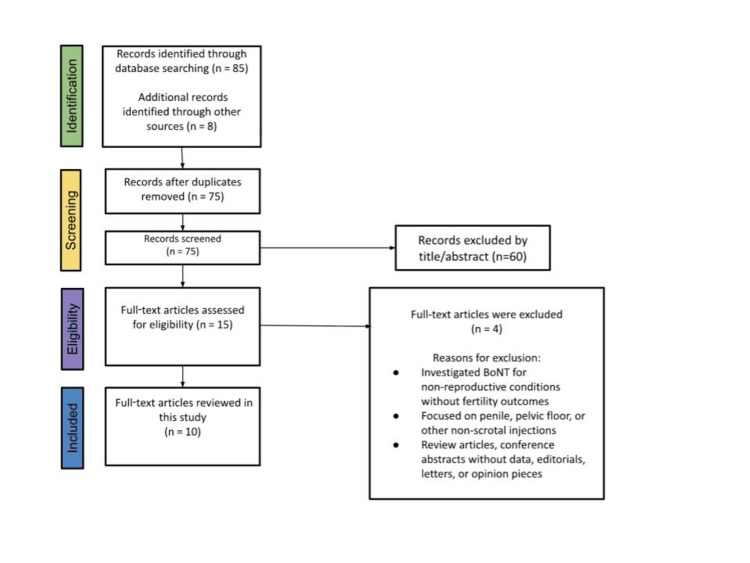
PRISMA flow diagram illustrating the identification, screening, eligibility assessment, and inclusion of studies evaluating scrotal or peritesticular botulinum toxin exposure and male reproductive outcomes.

The literature search and study selection process is summarized in Figure 1. A total of 93 records were identified, including 85 from database searches and eight from additional sources. After removal of duplicates, 75 records underwent title and abstract screening, of which 60 were excluded. Fifteen full-text articles were assessed for eligibility, and 10 studies met the inclusion criteria and were included in the qualitative synthesis.

The included studies comprised a heterogeneous body of evidence spanning preclinical animal toxicology and mechanistic studies as well as human case reports, case series, and clinical trials. The quality of evidence for human clinical studies is summarized in Table 1, and the quality of evidence for animal studies is summarized in Table 2, with fertility-relevant outcomes across studies synthesized in Table 3.

**Table 1:** Quality of evidence: human clinical studies

Study	Design / population	Exposure (BoNT dose and site)	Fertility outcomes measured	Risk of bias / key limitations	Quality appraisal tool used	Level of evidence (Oxford CEBM)	Overall quality of evidence for male fertility
Ramelli et al., 2020 [1]	Single case report; 44-year-old man requesting aesthetic scrotal relaxation	Intrascrotal BoNT-A to dartos ± cremaster (dose not clearly specified)	None (no semen, hormones, or testicular imaging)	Uncontrolled single case; short follow-up; subjective cosmetic outcome only; no fertility assessments	JBI Critical Appraisal Checklist for Case Reports	Level 5 (case report)	Very Low - does not assess fertility; only demonstrates feasibility and short-term safety in one patient
Mori et al., 2011 [2]	Case report; 26-year-old man with debilitating cremasteric synkinesia	100 U BoNT-A injected into bilateral cremasteric muscles over three sessions	None	Single patient; no control; focuses on pain/spasm scores; no semen or endocrine testing	JBI Checklist	Level 5	Very Low - provides symptom relief data only; no information on fertility
Ritter et al., 2006 [19]	Case report; 62-year-old man with cremaster synkinesias	15 MU BoNT-A (right) and 10 MU (left) into cremaster muscles	None	Single case; brief follow-up; no fertility endpoints; outcomes limited to synkinesia and pain	JBI Checklist	Level 5	Very Low - entirely neuromuscular outcome; fertility effects unknown
Khambati et al., 2014 [20]	Open-label pilot trial; men with chronic scrotal pain responsive to diagnostic cord block	100 U onabotulinumtoxinA injected in perispermatic cord block	None	Small sample; no control group; non-blinded; primary outcome is pain score; no reproductive evaluations	JBI Checklist for Case Series	Level 4 (case series / uncontrolled cohort)	Very Low - suggests symptomatic benefit but offers no data on spermatogenesis or hormones
Dockray et al., 2020 [21]	Randomized, double-blind, placebo-controlled trial; men with chronic scrotal pain	200 U onabotulinumtoxinA vs local anesthetic alone via spermatic cord block	None	Moderate RCT; adequate blinding; primary endpoint pain; no fertility or endocrine measures; relatively small N for safety rare events	Cochrane RoB 2.0	Level 1b (individual RCT)	Low (for fertility) - high-quality pain RCT but entirely indirect for fertility; lack of semen/hormone data limits inference

**Table 2:** Quality of evidence: animal toxicology studies

Study	Model / design	Exposure (BoNT dose and site)	Fertility outcomes measured	Risk of bias / key limitations	Quality appraisal tool used	Overall quality of evidence for male fertility*
Cakmak et al., 2003 [10]	Adult Wistar rats; mechanistic neurophysiology study	Single intracremasteric BoNT-A injection (dose not clearly reported)	None – only cremasteric compound muscle action potentials	Very small sample; no reproductive or hormonal endpoints; short follow-up; indirect to fertility	Not applicable (no fertility endpoints)	Very low - informative for cremasteric paralysis but provides no direct fertility data
Breikaa et al., 2014 [17] (mature rats)	Mature male rats; dose-response experimental toxicology	Intracremasteric BoNT-A 10–40 U/kg, repeated	Sperm count, motility, semen quality, testicular histology, testosterone, LDH-X	No explicit randomization/blinding; high doses relative to clinical use; single species; limited long-term recovery data	SYRCLE Risk of Bias Tool	Moderate - robust set of fertility endpoints, but methodological detail limited and exposure not directly clinically generalizable
FDA Botox® Reproductive Tox Study [18]	Adult male and female rats; GLP regulatory fertility study	IM BoNT-A 4, 8, 16 U/kg, repeated prior to and on mating	Fertility indices (mating, conception, litter parameters); testicular atrophy reported in associated tox data	Conducted to regulatory standards but only summarized in label; limited published methodological detail; no sperm parameters reported	OECD/GLP Standard Toxicology Evaluation	Moderate - relatively rigorous GLP design and clear fertility signal, but outcome reporting is coarse and indirect
Radhakrishnan et al., 2022 [22] (low-dose IM limb)	Ageing male BALB/c mice; controlled experimental study (n≈12; 6 BoNT vs 6 control)	Single IM injection 1 U/kg into vastus lateralis (limb)	Sperm count, motility, morphology, testicular histology, antioxidant markers	Small sample; limited detail on randomization/blinding; short follow-up; single species/strain	SYRCLE Risk of Bias Tool	Moderate - directly assesses spermatogenesis; internal validity reasonable but underpowered and single-study evidence
Breikaa et al., 2016 [23] (growing rats)	Immature male rats; experimental toxicology	Intracremasteric BoNT-A 10–40 U/kg, repeated	Sperm count, motility, testicular histology; germ-cell loss	Similar limitations as the 2014 study; immaturity model may exaggerate toxicity; short follow-up	SYRCLE Risk of Bias Tool	Low-moderate - consistent signal of testicular toxicity in a vulnerable model; risk of bias and indirectness lower confidence

**Table 3:** Summary of fertility-related outcomes across studies

Study	Model	BoNT dose / site	Sperm count	Motility	Morphology	Testicular histology	Hormones	Temperature	Overall effect
Ramelli et al., 2020 [1]	Human (aesthetic)	Dartos + cremaster	–	–	–	–	–	–	No fertility data
Mori et al., 2011 [2]	Human	Cremasteric injections	–	–	–	–	–	–	No fertility data
Cakmak et al., 2003 [10]	Rats	Intracremasteric BoNT-A	(Not clearly reported)	(Not reported)	–	–	–	–	Mechanistic only Suggests negative effect
Breikaa et al., 2014 [17] (mature rats)	Mature rats	10–40 U/kg intracremasteric	↓	↓	–	↓ (degeneration, fibrosis)	↓ (testosterone, LDH-X changes)	–	Deterioration
FDA Botox® Reproductive Tox Study [18]	Adult rats	Systemic IM, repeated	↓	–	–	↓	–	–	Deterioration
Ritter et al., 2006 [19]	Human	Cremasteric injections	–	–	–	–	–	–	Symptom relief of cremaster synkinesias; No fertility data
Khambati et al., 2014 [20]	Human	100 U Botox in Perispermatic cord	–	–	–	–	–	–	Symptom relief only
Dockray et al., 2020 [21]	Human (chronic scrotal pain)	200 U/kg onabotulinumtoxinA via spermatic cord block	–	–	–	–	–	–	No improvement vs control; no fertility data
Radhakrishnan et al., 2022 [22] (low-dose IM limb)	Ageing mice	1 U/kg, IM vastus lateralis	↑	↑	↑	↑ (improved seminiferous structure)	(no hormone measurements; ↑ antioxidant markers)	–	Improvement
Breikaa et al., 2016 [23] (growing rats)	Immature rats	10–40 U/kg intracremasteric	↓	↓	–	↓ (germ cell loss)	–	–	Deterioration

Overall, animal studies provided moderate-quality evidence for dose- and site-dependent effects of botulinum toxin on spermatogenesis and testicular structure, supported by direct measurement of sperm parameters and histopathology but limited by small sample sizes, short follow-up, and indirect clinical relevance. In contrast, human studies were uniformly rated as low or very low quality for fertility outcomes, as none prospectively assessed semen parameters, endocrine function, or reproductive endpoints; instead, outcomes were limited to symptom relief, aesthetic satisfaction, or neuromuscular effects. Even the single randomized controlled trial included was considered low quality for fertility inference due to the absence of reproductive outcome measures.

Together, Figure 1 and Tables 1-3 demonstrate that the current evidence base is small, methodologically heterogeneous, and heavily weighted toward animal data, with a marked absence of direct human fertility assessments despite increasing clinical and cosmetic use of scrotal or peritesticular botulinum toxin.

Characteristics of the Included Studies

Across the available literature, we identified a small but heterogeneous set of experimental and clinical reports that directly or indirectly inform the potential impact of scrotal, cremasteric, or peritesticular botulinum toxin exposure on male reproductive physiology. Ten key studies, spanning animal toxicology, mechanistic physiology, human case reports, and clinical pain-management trials, are summarized in Table 4.

**Table 4:** Characteristics of key studies evaluating scrotal, cremasteric, or peritesticular botulinum toxin exposure and male reproductive outcome

Study (link)	Study type	Model / population	BoNT serotype and approx. dose	Injection site	Sperm parameters measured?	Testicular histology?	Hormones measured?	Temp. measured?	Symptom / clinical outcomes	Key fertility-relevant findings	Adverse effects reported
Ramelli et al., 2020 (Progès en Urologie) (ScienceDirect) [1]	Human case report (aesthetic)	44-year-old man seeking “scrotal rejuvenation”	BoNT-A; total ~80 U, diluted and divided	Intrascrotal: dartos and cremaster muscles	No	No	No	No	Aesthetic outcomes; patient satisfaction, scrotal appearance	Procedure technically feasible; authors emphasize that infertility risk is theoretical but unknown, and discourage use in men desiring future fertility	No acute adverse effects reported; patient requested repeat treatment; long-term fertility not assessed
Mori et al., 2011 (Urology) (Gold Journal) [2]	Human case report (functional)	Adult man with debilitating cremasteric synkinesia	BoNT-A; 100 U per session, 3 staged injections	Bilateral cremaster muscles	No	No	No	No	Marked reduction in painful cremasteric spasms and testicular retraction; improved quality of life	No semen or hormonal data; authors cite animal data suggesting potential testicular effects and note lack of fertility information	No short-term complications; no reported changes in testicular size or consistency
Cakmak et al., 2003 (Urol Res) (PubMed) [10]	Animal mechanistic	Adult Wistar rats	BoNT-A; single intramuscular dose (dose not fertility-focused)	Unilateral cremaster muscle	No	No	No	No	N/A	Demonstrated significant and sustained paralysis of cremaster muscle (↓ CMAP amplitude), supporting BoNT-A as a potential non-surgical option for retractile testes	No testicular toxicity endpoints measured; no major systemic adverse events reported
Breikaa et al., 2014 (Toxicol Appl Pharmacol) (PubMed) [17]	Animal toxicology	Adult male rats	OnabotulinumtoxinA; 10, 20, 40 U/kg; 3× over 2 weeks	Bilateral intracremasteric	Yes – count, motility, semen quality	Yes – seminiferous tubule structure, fibrosis	Yes – testosterone, LDH-X, acid phosphatase	No	N/A (animal)	Dose-dependent reduction in mature sperm, impaired semen quality, and inhibited spermatogenesis; suggests decreased fertility risk at higher doses	Histopathological seminiferous tubule changes, increased connective tissue; no major systemic toxicity reported
FDA Botox® label, 2021 (FDA Access Data) [18]	Regulatory reproductive toxicology	Juvenile rats	OnabotulinumtoxinA; 8, 16, 24 U/kg IM every other week	Systemic intramuscular (non-scrotal)	Yes – fertility/mating indices	Yes – degeneration of seminiferous tubules	Not detailed (standard tox panel)	No	N/A	Mid- and high-dose groups showed impaired fertility and testicular histopathology; effects partly reversible after dosing cessation	Degeneration of seminiferous tubules, reduced fertility; bone changes due to limb disuse
Ritter et al., 2006 (Mov Disord) (PubMed) [19]	Human case report (functional)	62-year-old man with bilateral cremaster synkinesias after laparotomy	BoNT-A; dose not fertility-focused (per-muscle injections)	Bilateral cremaster muscles	No	No	No	No	Resolution of cremasteric synkinesias refractory to oral meds	Demonstrates feasibility of targeted cremasteric BoNT-A; no assessment of fertility or endocrine function	No significant adverse events; no reported impact on testicular volume or pain
Khambati et al., 2014 (J Sex Med) (PubMed) [20]	Human open-label trial	18 men with chronic scrotal pain	OnabotulinumtoxinA; 100 U in local anesthetic	Perispermatic / spermatic cord nerve block at external inguinal ring	No	No	No	No	Significant reduction in pain scores and scrotal tenderness up to 3 months; improved Chronic Epididymitis Symptom Index	Provides indirect evidence on repeated peritesticular exposure without obvious acute testicular toxicity, but fertility not evaluated	Transient local discomfort; no serious adverse events or clinically apparent testicular atrophy
Dockray et al., 2020 (J Urol) (AUA Journals) [21]	Human randomized double-blind trial	Men with chronic scrotal pain	Onabotulinum toxin A; 100 U vs placebo	Perispermatic / spermatic cord nerve block	No	No	No	No	Trial found no significant advantage of BoNT-A over placebo for pain at 1 month	Confirms that perispermatic BoNT-A at this dose did not reveal overt clinical testicular toxicity, but fertility outcomes were not collected	Mild local adverse events only; no serious or fertility-related adverse events reported
Radhakrishnan et al., 2022 [22]	Animal experiment	Ageing male mice	BoNT-A, 1 U/kg	Distal IM (vastus lateralis)	Yes – count, motility, morphology	Yes	Antioxidant markers (SOD, CAT, GSH, GPx) – not sex hormones	No	Not applicable	Significant improvement in spermatogenesis, ↑ sperm count, motility, morphology; ↑ antioxidant activity	None
Breikaa et al., 2016 (J Appl Toxicol) (PubMed) [23]	Animal toxicology	Immature male rats (growing testes)	OnabotulinumtoxinA; 10, 20, 40 U/kg; 3× over 2 weeks	Bilateral intracremasteric	Yes – fertility indices, sperm maturation	Yes – maturing testicular structures	Limited – selected markers; antisperm antibodies	No	N/A	OnabotulinumtoxinA adversely affected sperm maturation and fertility indices; increased apoptosis and antisperm antibodies at higher doses	Degeneration of seminiferous epithelium and increased apoptosis; no severe systemic toxicity described

Five studies were preclinical, using juvenile or adult rats to evaluate testicular structure and function after repeated botulinum toxin exposure, typically via bilateral intracremasteric or intramuscular injections. Two of these were hypothesis-driven toxicology experiments by Breikaa et al. in mature and growing rats, respectively, focusing on spermatogenesis and testicular histology [17]. Two additional reproductive-toxicity datasets came from regulatory submissions for onabotulinumtoxinA (Botox) and prabotulinumtoxinA (Nuceiva), which reported seminiferous tubule degeneration and impaired fertility in male rats at higher doses [18]. A fifth preclinical study by Cakmak et al. examined the functional impact of cremasteric botulinum toxin injections on cremaster muscle activity as a model for retractile testes [10]. Five studies involved human participants. These included a single aesthetic intrascrotal BoNT-A case report by Ramelli et al. (cremaster and dartos injections), two case reports of intracremasteric BoNT-A for debilitating cremasteric synkinesia, and two clinical trials-one randomized, placebo-controlled study by Dockray et al. and one open-label trial using onabotulinumtoxinA spermatic cord or inguinal nerve blocks for chronic scrotal pain [1,2,19,20,21]. None of the human studies prospectively measured semen parameters or endocrine outcomes, and fertility endpoints were either absent or inferred only at a theoretical level.

Injection Sites, Serotypes, and Dosing

Most preclinical work used onabotulinumtoxinA (BoNT-A) administered bilaterally into the cremaster muscle at doses of 10-40 U/kg, repeated three times over two weeks, allowing dose-response evaluation of testicular toxicity [17]. Regulatory rat studies used repeated systemic intramuscular injections (non-scrotal) but provide important contextual data on dose thresholds associated with testicular histopathology and reduced fertility [18]. For cremasteric synkinesia, case reports describe administering 100 units of onabotulinumtoxinA directly into the cremaster muscle per treatment session, resulting in marked reduction of spasms and testicular retraction [2,19]. Chronic scrotal pain trials typically used 100 U onabotulinumtoxinA diluted in local anesthetic and injected around the spermatic cord at the external inguinal ring (perispermatic/peritesticular nerve block) [20]. For aesthetic applications, published reports of intrascrotal BoNT-A injections typically emphasize technique rather than specific dosing, and the single peer-reviewed aesthetic case report by Ramelli et al. (2020) does not specify a total unit dose [1].

Populations and Outcomes Measured

Preclinical toxicology studies systematically assessed sperm parameters (count, motility, maturation indices), testicular histology, and testosterone or related biochemical markers, with some also evaluating oxidative/inflammatory status and antisperm antibody formation [17,22]. None of the animal studies directly measured scrotal or testicular temperature, although changes in cremaster tone and spermatogenic disruption provide indirect insight into thermoregulatory risk. In humans, outcomes were almost exclusively symptomatic; they only included aesthetic satisfaction, reduction in testicular retraction or synkinesia, and pain scores (visual analogue scale, symptom indices), with no prospective semen analysis or hormonal profiling [1,2,19]. 

Key Fertility-Relevant Findings

In mature and growing rats, repeated intracremasteric onabotulinumtoxinA injections caused dose-dependent impairment of spermatogenesis, reduced sperm counts, delayed sperm maturation, and histopathological changes such as seminiferous tubule degeneration and increased apoptosis, while serum testosterone was often preserved [17,23]. Regulatory juvenile rat studies similarly reported impaired male fertility and degeneration of seminiferous tubules at mid-to-high systemic doses, with partial reversibility after cessation [18,23]. By contrast, the small human scrotal/cremasteric case series and chronic pain trials did not document any clear fertility signal, but they also did not systematically look for it; authors frequently acknowledged the theoretical risk of impaired fertility and recommended caution in men desiring future paternity [1,10]. Overall, the evidence base is dominated by animal data indicating potential spermatotoxicity at higher or repeated doses, while human data remain limited to symptomatic outcomes with minimal reproductive monitoring.

Mechanistic Studies

The primary proposed biological mechanism of intra-scrotal or peri-testicular injection of BoNT-A is neuromuscular blockade at the level of the smooth-muscle fibers of the Dartos muscle in the scrotal wall and the Cremaster muscle surrounding the testis. Specifically, BoNT-A acts by inhibiting acetylcholine release at cholinergic nerve terminals, thus preventing contraction of these muscles [24]. In one animal study, intramuscular BoNT-A into the cremaster of rats significantly reduced compound muscle action potentials 45 days after injection (mean 0.44 ± 0.25 µV vs. baseline ~3.25 µV), demonstrating effective paralysis of cremasteric contractility [10]. By relaxing the dartos and cremaster muscles, the scrotal sac tends to hang lower and less tightly, which is the aesthetic basis for the so-called “scrotox” procedure as well as the functional hypothesis of altered thermoregulation [1].

From a thermoregulatory standpoint, the dartos and cremaster muscles both play critical roles in maintaining testicular temperature within the narrow range required for optimal spermatogenesis: the dartos modifies surface area of the scrotal skin (wrinkling or smoothing), and the cremaster modulates elevation or descent of the testes closer to or farther from the body [9,25,26,27,28]. Interfering with this musculature via BoNT-A might thus alter the cooling capacity of the scrotum: for example, a flaccid cremaster may allow testes to hang colder too far from the body or conversely may reduce the ability to elevate in colder ambient temperatures [26,28,29]. Some commentary in the aesthetic literature has raised the concern that relaxing these muscles may impair thermoregulation and thereby pose a theoretical risk to spermatogenesis, although clinical human fertility data are lacking [9,30].

In summary, mechanistic studies (largely animal or observational in nature) support the idea that BoNT-A applied to the scrotal musculature can effectively suppress contractile function of the dartos and cremaster muscles by neuromuscular blockade [10, 31]. Evidence from animal models confirms altered muscle electrical activity and morphology after injection. Because those muscles are integral to testicular thermoregulation, relaxing them could theoretically lead to altered intratesticular temperature and potentially impact sperm function or production, although human data remain sparse and definitive fertility outcomes are not yet established [1,17].

Dose- and Site-Dependent Effects of BoNT-A on Spermatogenesis in Animal Models

Low-dose BoNT-A animal studies using mild systemic intramuscular administration, rather than direct scrotal or cremasteric injection, have reported neutral or even favorable effects on spermatogenesis in ageing models. In ageing mice, a single intramuscular injection of 1 U/kg BoNT-A into a limb muscle significantly increased total sperm count and progressive motility compared with age-matched controls, accompanied by more regular seminiferous tubule architecture and increased numbers of spermatogenic cells [22]. These effects were associated with increased activities of key testicular antioxidant enzymes (SOD, catalase, GPx, GSH) and reduced oxidative stress markers, suggesting a potential protective or anti-ageing effect on testicular function [22].

By contrast, high-dose or directly intracremasteric BoNT-A administration consistently impairs testicular structure and function in rodent models [17]. In mature rats, repeated intracremasteric injections of Botox® at 10-40 U/kg reduced the gonadosomatic index, disrupted seminiferous tubule architecture with fibrotic separation, impaired semen quality, and reduced mature sperm populations [17]. Comparable dosing in growing rats resulted in decreased sperm count and semen quality, delayed sperm maturation on DNA ploidy analysis, and increased apoptosis markers, with serum testosterone remaining largely unchanged [23].

Collectively, these findings demonstrate a clear dose- and site-dependent effect of BoNT-A on male fertility: very low systemic intramuscular doses distant from the testes may exert neutral or protective effects, whereas repeated high-dose injections delivered adjacent to the testes are overtly spermatotoxic [17,22,23]. These data caution against extrapolating low-dose systemic findings to scrotal or cremasteric BoNT applications and underscore the need for careful consideration of dose, injection site, and cumulative exposure when evaluating potential fertility risks.

Human Studies

Chronic orchialgia/cremasteric reflex studies: Human studies using botulinum toxin for chronic orchialgia or exaggerated cremasteric reflex primarily arise from small case reports and pilot trials addressing disabling testicular pain or hyperactive cremasteric muscle contractions. These studies commonly involve direct intracremasteric injection or perispermatic nerve-block techniques aimed at reducing painful spasms or retraction episodes [2,19,20].

Across these reports, botulinum toxin reliably decreases cremasteric hyperactivity and improves pain scores or functional symptoms, which is an effect attributed to reduced neuromuscular excitability of the cremaster muscle [2]. However, none of these studies include semen analyses, endocrine assessments, or any formal reproductive endpoints, leaving fertility outcomes entirely uncharacterized [2,20]. Reported adverse effects across these clinical experiences are minimal and transient, generally limited to mild procedural discomfort without evidence of testicular atrophy or structural harm [20].

Cosmetic scrotox studies: Cosmetic “scrotox” patients typically pursue treatment to achieve smoother scrotal skin, reduced wrinkling, or improved perceived testicular relaxation rather than to address pain or functional pathology [1]. Case reports describe noticeable relaxation of the dartos and cremaster muscles following intrascrotal botulinum toxin, producing a lower-hanging and less contracted scrotal appearance [1,10]. Patients in these cosmetic reports generally report high satisfaction with perceived improvements in scrotal smoothness and comfort [1,7].

Although cosmetic scrotox studies have not assessed semen parameters, hormonal profiles, or testicular thermoregulation, leaving reproductive outcomes undefined, the cosmetic urology literature consistently raises theoretical fertility concerns related to potential disruption of scrotal thermoregulation despite the absence of empirical human data [1,17].

Trends in Scrotox Interest and Utilization

Accurate prevalence estimates for scrotal botulinum toxin injection (“scrotox’) are currently unavailable due to the absence of procedure-specific coding, limited reporting within the medical literature, and the predominance of self-paid cosmetic administration. Existing peer-reviewed studies primarily evaluate botulinum toxin for chronic scrotal content pain, with published cohorts generally comprising several dozen patients (e.g., 22-44 patients per series) [20]. By contrast, potential aesthetic use is indirectly suggested by emerging indicators outside clinical data streams. Reported search query analyses have identified approximately 40,000 searches for “scrotox” in recent years [32], and commercial scans have identified a modest number of aesthetic and urologic practices publicly marketing scrotal botulinum toxin services across major metropolitan centers [33].

Despite suggestive indicators of increasing awareness, the scientific literature remains limited, with only eight to 10 publications directly addressing the use of scrotal botulinum toxin in therapeutic or aesthetic contexts [34,35]. The discrepancy between public visibility and scarce clinical documentation suggests a developing trend without corresponding epidemiological clarity. Current evidence, therefore, remains insufficient to characterize the magnitude of aesthetic utilization, long-term outcomes, or safety in non-therapeutic populations. Standardized procedural reporting, coding distinctions, and population-based data will be necessary to delineate true prevalence, clinical indications, and risk profiles associated with aesthetic scrotal botulinum. 

Discussion 

Current mechanistic, animal, and limited clinical evidence indicate that scrotal or peritesticular exposure to botulinum toxin can influence male reproductive physiology. However, data remain insufficient to support clinical guidance. Across experimental models, botulinum toxin consistently induces neuromuscular blockade of the dartos and cremaster muscles, which are essential for maintaining the thermoregulatory environment required for spermatogenesis [10,17]. Low-dose systemic injections in ageing animals have been associated with improved sperm quality and seminiferous organization, potentially via reduced oxidative stress [23]. In contrast, high-dose or locally targeted injections reliably impair spermatogenic function and produce structural degeneration within seminiferous tubules [17,22]. These findings demonstrate a dose- and site-specific response, highlighting a mechanistic evaluation of thermoregulatory disruption. 

Mechanistically, paralysis of the cremaster and dartos muscles may disrupt testicular thermoregulation, which depends on testicular elevation and modulation of scrotal surface area [10,36]. Spermatogenesis requires intratesticular temperatures 2-6°C below core body temperature [36,37], so small changes in heat dissipation may have physiologic effects. Repeated intracremasteric injections produce seminiferous epithelial degeneration and reduced germ-cell populations despite relative preservation of circulating testosterone [17,23]. However, no model has directly measured intratesticular or scrotal temperature following botulinum toxin exposure, leaving this mechanism unverified.

Toxicological data demonstrate a dose- and site-dependent profile in which systemic exposure elicits biologically distinct effects from targeted scrotal administration. Low-dose systemic injections distant from the testes improve sperm quality and seminiferous organization in ageing mice [23]. In contrast, repeated intracremasteric injections at 10-40 U/kg consistently induce fibrosis, germ-cell apoptosis, and impaired sperm maturation in both adult and immature rat models [17,22]. These contrasting outcomes underscore that injection dose, anatomical site, and cumulative exposure are critical determinants of testicular risk, reinforcing the need for standardized protocols in human scrotox applications. Although some animal studies describe partial reversibility of testicular injury following cessation of exposure [22,34], neither the extent nor the durability of recovery is defined. Furthermore, existing models evaluate only acute spermatogenic indices, leaving long-term fertility outcomes and recovery trajectories unresolved. 

Human evidence is restricted to isolated case reports and small interventional studies not designed to assess reproductive physiology. These investigations primarily employ botulinum toxin for chronic orchialgia or exaggerated cremasteric reflex, where clinical improvement results from chemodenervation of cholinergic motor terminals via SNAP-25 cleavage, producing sustained paralysis of the cremaster muscle [2,7]. Although this innervation suppresses pathological testicular retraction, none of these studies measure fertility-relevant endpoints, including semen parameters, gonadal endocrine function, or testicular histopathology [7,21]. Cosmetic applications apply the same biochemical mechanism to the dartos muscle to reduce scrotal tension, yet similarly report only subjective alterations in appearance and omit evaluation of thermoregulatory function, including dartos-mediated modulation of scrotal surface area and compensatory changes in pampiniform plexus countercurrent heat exchange [20]. Pain management studies also report no clinically evident testicular injury following perispermatic or inguinal injection [2]; however, the lack of chemodenervation-specific monitoring, such as intratesticular temperature mapping, vascular heat exchange assessment, or hormonal profiling, prevents physiologic or mechanistic inference regarding reproductive safety. 

By contrast, animal studies demonstrate a dose-dependent toxicity profile, particularly when higher doses of botulinum toxin are injected near the testes or directly into the cremaster muscle. Experimental intracremasteric injections of 10-40 U/kg in rats produce seminiferous tubule degeneration, reduced sperm populations, and impaired spermatogenesis [17]. These doses and exposure conditions in animal models are not directly extrapolable to those used in reported human cases, particularly in aesthetic applications, but they remain relevant for identifying potential biological hazards under high local exposure conditions. Growing rat models show delayed sperm maturation, increased apoptosis, and structural compromise of germinal epithelium under similar dosing conditions [23]. These toxicological effects raise concern because both the dartos and cremaster muscles play central roles in scrotal thermoregulation, which is essential for maintaining testicular temperature within the narrow range required for sperm production [26]. Even though human reports have not documented overt fertility harm, the combination of animal data and established thermophysiology underscores thermoregulatory disruption as a plausible risk that warrants caution and targeted study in future clinical research [1,10,17].

Taken together, current evidence highlights substantial knowledge gaps that require targeted investigation. No human study has prospectively evaluated semen parameters, endocrine function, or testicular temperature following scrotal or cremasteric botulinum toxin injection, despite the procedure’s mechanistic potential to affect fertility [2,34]. Dosing protocols lack standardization, and injection techniques vary widely across aesthetic and therapeutic applications, preventing meaningful dose-response assessment [10,34]. In addition, animal studies demonstrate dose and site-dependent testicular toxicity without direct thermoregulation measurements, even though elevated testicular temperatures impair spermatogenesis [17,23,38]. Finally, neither animal nor human literature addresses long-term reproductive outcomes, including recovery after repeated exposure or effects on fertility potential [17,20]. Addressing these gaps will require controlled dose finding studies, standardized injection techniques, incorporation of thermophysiological monitoring, and longitudinal reproductive follow-up. Until such evidence exists, clinicians should exercise caution when offering scrotox to men who desire future fertility, recognizing that mechanistic risk is plausible and human safety data remain absent [17,34].

## Conclusions

Scrotal botulinum toxin injections remain inadequately studied with respect to male reproductive health, and current evidence is insufficient to determine whether they pose benefit, harm, or no effect on fertility. Animal research demonstrates a precise dose and site-dependent pattern: high-dose or cremasteric injections disrupt spermatogenesis and damage seminiferous architecture, whereas low systemic doses distant from the testes may improve sperm quality in aging models. Mechanistic studies further suggest that paralysing the dartos and cremasteric muscles may alter thermoregulatory control, a critical determinant of normal spermatogenesis. Still, no study has yet confirmed this pathway through direct temperature measurements in humans or animals. Human data are limited to aesthetic and pain management case reports and small trials that do not assess semen parameters, hormonal profiles, or long-term reproductive outcomes. Given these significant knowledge gaps, including unmeasured thermophysiological effects and undefined dose thresholds, the reproductive safety of scrotal botulinum toxin cannot be assumed. Until control clinical trials incorporate standardized dosing, thermoregulation metrics, and fertility endpoints, clinicians should avoid scrotox in men seeking to preserve fertility. 

## Appendices

Appendix A: database search strategies

Overview

This appendix provides the exact, reproducible electronic search strategies used for all databases included in the systematic review. Searches were designed to identify studies evaluating botulinum toxin exposure involving the scrotum, dartos, cremaster, spermatic cord, or peritesticular region, as well as predefined regulatory male fertility datasets and a restricted contextual body of systemic botulinum toxin literature assessing spermatogenesis outcomes. All searches were limited to English-language publications.

The final search date for all databases was January 5, 2026.

PubMed/MEDLINE (searched December 5, 2025)

Query 1 (scrotal/cremasteric/peritesticular exposure):

(("Botulinum Toxins"[Mesh] OR botulinum toxin*[tiab] OR onabotulinumtoxinA[tiab] OR prabotulinumtoxinA[tiab] OR Botox[tiab] OR Nuceiva[tiab]) AND (scrot*[tiab] OR "scrotal"[tiab] OR dartos[tiab] OR cremaster*[tiab] OR "spermatic cord"[tiab] OR peritesticular[tiab] OR orchialgia[tiab] OR "chronic scrotal pain"[tiab] OR "cremasteric synkinesia"[tiab] OR retractile[tiab])) AND (english[lang])

Query 2 (restricted contextual systemic BoNT-A spermatogenesis evidence):

((botulinum toxin*[tiab] OR onabotulinumtoxinA[tiab] OR "Botulinum Toxins"[Mesh]) AND (spermatogenesis[tiab] OR sperm*[tiab] OR testicular[tiab] OR testis[tiab]) AND (ageing[tiab] OR aging[tiab] OR "aged"[tiab] OR "aging"[tiab])) AND (mice[tiab] OR mouse[tiab] OR murine[tiab]) AND (english[lang])

Embase (Elsevier Embase.com; searched December 5, 2025)

Query 1 (scrotal/cremasteric/peritesticular exposure):

('botulinum toxin'/exp OR botulinum toxin*:ti,ab OR onabotulinumtoxinA:ti,ab OR prabotulinumtoxinA:ti,ab OR Botox:ti,ab OR Nuceiva:ti,ab) AND (scrot*:ti,ab OR dartos:ti,ab OR cremaster*:ti,ab OR 'spermatic cord':ti,ab OR peritesticular:ti,ab OR orchialgia:ti,ab OR 'chronic scrotal pain':ti,ab OR 'cremasteric synkinesia':ti,ab OR retractile:ti,ab) AND [english]/lim

Query 2 (restricted contextual systemic BoNT-A spermatogenesis evidence):

('botulinum toxin'/exp OR botulinum toxin*:ti,ab OR onabotulinumtoxinA:ti,ab) AND (spermatogenesis:ti,ab OR sperm*:ti,ab OR testis:ti,ab OR testicular:ti,ab) AND (ageing:ti,ab OR aging:ti,ab OR aged:ti,ab) AND (mouse:ti,ab OR mice:ti,ab OR murine:ti,ab) AND [english]/lim

Scopus (searched December 5, 2025)

Query 1 (scrotal/cremasteric/peritesticular exposure):

TITLE-ABS-KEY (( "botulinum toxin" OR onabotulinumtoxinA OR prabotulinumtoxinA OR botox OR nuceiva ) AND ( scrot* OR dartos OR cremaster* OR "spermatic cord" OR peritesticular OR orchialgia OR "chronic scrotal pain" OR "cremasteric synkinesia" OR retractile )) AND ( LIMIT-TO ( LANGUAGE, "English" ))

Query 2 (restricted contextual systemic BoNT-A spermatogenesis evidence):

TITLE-ABS-KEY ( ( "botulinum toxin" OR onabotulinumtoxinA ) AND ( spermatogenesis OR sperm* OR testis OR testicular ) AND ( ageing OR aging OR aged ) AND ( mouse OR mice OR murine ) ) AND ( LIMIT-TO ( LANGUAGE, "English" ) )

Additional Sources

In addition to database searches, we manually screened reference lists of included studies and relevant reviews. We also conducted targeted retrieval of publicly available regulatory reproductive-toxicity documentation from the U.S. Food and Drug Administration (FDA) and the European Medicines Agency (EMA) for onabotulinumtoxinA (BOTOX®) and prabotulinumtoxinA (Nuceiva®).
